# Combined Perfusion and Permeability Imaging Reveals Different Pathophysiologic Tissue Responses After Successful Thrombectomy

**DOI:** 10.1007/s12975-020-00885-y

**Published:** 2021-01-11

**Authors:** Arne Potreck, Matthias A. Mutke, Charlotte S. Weyland, Johannes A. R. Pfaff, Peter A. Ringleb, Sibu Mundiyanapurath, Markus A. Möhlenbruch, Sabine Heiland, Mirko Pham, Martin Bendszus, Angelika Hoffmann

**Affiliations:** 1 Department of Neuroradiology, Heidelberg University Hospital, INF 400, 69120 Heidelberg, Germany; 2 Department of Neurology, Heidelberg University Hospital, Heidelberg, Germany; 3 Division of Experimental Radiology, Department of Neuroradiology, Heidelberg University Hospital, Heidelberg, Germany; 4 Department of Neuroradiology, Würzburg University Hospital, Würzburg, Germany; 5 University Institute of Diagnostic and Interventional Neuroradiology, University Hospital Bern, Inselspital, University of Bern, Bern, Switzerland

**Keywords:** Permeability imaging, Perfusion imaging, Mechanical thrombectomy, Secondary stroke injury, Hyperperfusion

## Abstract

Despite successful recanalization of large-vessel occlusions in acute ischemic stroke, individual patients profit to a varying degree. Dynamic susceptibility-weighted perfusion and dynamic T1-weighted contrast-enhanced blood-brain barrier permeability imaging may help to determine secondary stroke injury and predict clinical outcome. We prospectively performed perfusion and permeability imaging in 38 patients within 24 h after successful mechanical thrombectomy of an occlusion of the middle cerebral artery M1 segment. Perfusion alterations were evaluated on cerebral blood flow maps, blood-brain barrier disruption (BBBD) visually and quantitatively on *k*_trans_ maps and hemorrhagic transformation on susceptibility-weighted images. Visual BBBD within the DWI lesion corresponded to a median *k*_trans_ elevation (IQR) of 0.77 (0.41–1.4) min^−1^ and was found in all 7 cases of hypoperfusion (100%), in 10 of 16 cases of hyperperfusion (63%), and in only three of 13 cases with unaffected perfusion (23%). BBBD was significantly associated with hemorrhagic transformation (*p* < 0.001). While BBBD alone was not a predictor of clinical outcome at 3 months (positive predictive value (PPV) = 0.8 [0.56–0.94]), hypoperfusion occurred more often in patients with unfavorable clinical outcome (PPV = 0.43 [0.10–0.82]) compared to hyperperfusion (PPV = 0.93 [0.68–1.0]) or unaffected perfusion (PPV = 1.0 [0.75–1.0]). We show that combined perfusion and permeability imaging reveals distinct infarct signatures after recanalization, indicating the severity of prior ischemic damage. It assists in predicting clinical outcome and may identify patients at risk of stroke progression.

**Supplementary Information:**

The online version contains supplementary material available at 10.1007/s12975-020-00885-y.

## Introduction

Mechanical thrombectomy is an effective treatment for patients with acute ischemic stroke due to large vessel occlusion. Early and complete recanalization can be achieved in upwards of 70% of patients and macrovascular angiographic recanalization is an important variable required for favorable outcomes [1–6]. However, even despite full recanalization of large vessel occlusions, adverse outcomes are still regularly observed. This discrepancy is not fully explained by the extent of early brain infarction at baseline before recanalization [7]. Final infarct size on follow-up imaging also is not found to completely explain the treatment effect of endovascular stroke therapy [7, 8]. In an attempt to better explain why reperfusion does not consistently lead to good clinical outcomes, evaluating brain microvascular and tissue response after ischemia and reperfusion may be suited to predict tissue fate.

Advanced imaging methods such as perfusion and permeability MR imaging can visualize downstream brain tissue response after upstream macrovascular recanalization. The body of literature assessing the post-reperfusion period of acute stroke is growing, but the pathophysiology after reperfusion and its implications in clinical outcomes are still a topic of debate. While hypoperfusion has been associated with infarct expansion after recanalization, hyperperfusion was once considered a hallmark of successful recanalization [9]. In a study of 100 stroke patients, hyperperfusion as measured by perfusion MR imaging was associated with improved clinical outcomes when compared to patients who displayed hypoperfusion [10]. These results are thought to be due to good arterial collateralization, penumbral salvage, and favorable infarct topography [11]. However, hyperperfusion has also been associated with hemorrhagic transformation and prolonged disturbance of consciousness [12–14]. With adverse outcomes noted with both hypo- and hyperperfusion after revascularization, it appears that both perfusion patterns indicate a certain degree of ischemic damage [15].

To better understand the underlying pathophysiology of cerebral perfusion after ischemic stroke, taking into account the microvascular response and blood-brain barrier integrity may contribute important information on stroke progression. Oxidative stress and free radical production, impairment of the neurovascular unit with blood-brain barrier disruption (BBBD) early, and inflammatory changes later after stroke are known to play a pivotal role in post-ischemic injury [16]. In our study, we combine dynamic T2*-weighted perfusion and dynamic T1-weighted contrast-enhanced permeability imaging in patients after successful mechanical thrombectomy to uncover possible patterns and combinations of altered cerebral perfusion and radiologic BBBD. We further examined their usefulness as predictive factors for early and late neurological outcomes.

## Materials and Methods

### Ethics Statement

All patients gave informed written consent. The study was approved by the ethics committee of Heidelberg University and conducted according to the principles expressed in the Declaration of Helsinki.

### Study Design

In this single-center, observational prospective imaging study, an MRI exam was acquired within 1 day after stroke onset from May 2017 until June 2019 in 44 patients. Inclusion criterion was the occlusion of an MCA M1 segment and successful mechanical thrombectomy (mTICI 2b–3). Imaging data from 6 patients had to be excluded due to severe motion artifacts and/or due to an incomplete MRI protocol. Out of the included 38 patients, 20 patients received i.v. tPA prior to thrombectomy. On admission, a CT was acquired and ASPECTS was determined. MR imaging performed at day 1 was compared to early and late neurological outcomes. Early clinical outcome was assessed by NIHSS at discharge with an NIHSS of ≤ 2 defined as an excellent favorable early clinical outcome [17]. Late neurological outcome was assessed by a mRS score at 3 months after stroke (mRS90) by an independent neurologist by telephone interrogation, blinded to the results of this study. Favorable clinical outcome was defined as functional independence (mRS90 0–2) or mRS90 = 3 in patients with pre-stroke mRS=3.

### Image Acquisition and Post-processing

Imaging was performed at 3-T MRI systems (Magnetom Verio/Trio TIM/Prismafit; Siemens Healthcare, Erlangen, Germany) with a 12-channel head matrix coil. The imaging protocol included diffusion-weighted imaging (EPI sequence, repetition and echo time (TR/TE) = 5300 ms/90 ms, flip angle = 90°, slice thickness (ST) = 5 mm, *b-*values = 0, 1200 s/mm^2^); 2D T2-weighted and 2D FLAIR-weighted imaging (TR = 5000 ms/8500 ms, TE = 85 ms/133 ms, ST = 5 mm); susceptibility-weighted imaging (TR = 27 ms, TE = 20 ms, ST = 2.5 mm); in 32 out of 38 patients, isotropic 3D T1-MPRAGE imaging (TR = 1800 ms, TE = 3.7 ms, ST = 1 mm); dynamic contrast-enhanced (DCE) imaging including T1 mapping (TR = 4.5 ms, TE = 1.74 ms, FA = 10°, ST = 5 mm, 26 slices, 60 repetitions, acquisition time = 4.5 min, 0.1 mmol/kg Gd-DOTA (Dotarem®) after 3–5 repetitions, variable FAs for T1 mapping = 2, 5, 8, 10, and 15°); and dynamic susceptibility contrast (DSC) imaging (TR/TE = 2220 ms/36 ms, flip angle = 90°, ST = 5 mm, 25 slices, 75 repetitions, 0.1 mmol/kg Gd-DOTA (Dotarem®) after 3–5 repetitions).

All sequences were co-registered to an individual standard space by linear (for FLAIR, T2, and DCE imaging) or non-linear (for DSC and DW imaging) registration using FMRIB’s Linear Image Registration Tool with either mutual information as cost function or boundary-based Epi-Registration [18, 19]. If available, registration was carried out to the 3D isotropic T1-MPRAGE imaging, else to 2D FLAIR imaging. The infarct core was manually segmented by two experienced readers (AP, MM) on DWI using ITK-SNAP [20]. Cerebral blood flow (CBF) maps derived from DSC-PWI were calculated with the FDA-approved Olea Sphere® perfusion (DSC) plug-in (Olea Medical®, La Ciotat, France) and the mean relative regional CBF (rrCBF) was calculated by normalizing the mean rCBF within the infarct lesion to the mean rCBF of the contralateral, healthy hemisphere. In addition to quantitative assessment, perfusion maps were visually assessed and classified into predominantly hypoperfused infarct core, predominantly hyperperfused infarct core, and unaffected perfusion (describing a visual perfusion pattern comparable to the visually corresponding region in the contralateral healthy hemisphere). We chose this visual approach as it allows to better account for physiological perfusion differences depending on the affected tissue compartment; e.g., white matter shows physiologically lower rrCBF compared to gray matter.

*K*_trans_ maps derived from motion-corrected DCE images (processed with MCFLIRT [19]) were calculated with the ImageJ plug-in TOPPCAT (T1-weighted perfusion imaging parameter calculation toolkit, free software by the Daniel P. Barboriak Laboratory (sites.duke.edu/dblab/toppcat/)) using a Patlak model [21]. *K*_trans_ maps were assessed quantitatively, as well as classified visually for BBBD within the infarct core. Hemorrhagic transformation (HT) on SWI was graded according to ECASS2 criteria [22], with HI1 defined as small petechiae along the margins of the infarct; HI2, as confluent petechiae within the infarcted area but no space-occupying effect; PH1, as blood clots in ≤ 30% of the infarcted area with some slight space-occupying effect; and PH2, as blood clots in > 30% of the infarcted area with a substantial space-occupying effect. Visual classification was carried out blinded to clinical data by three experienced readers (AH, AP, MM). Consensus rating was reached in case of disagreement.

### Statistical Analysis

Statistical analysis was performed with R* (The R Project for Statistical Computing, V3.1.2). Agreement of visual classification and quantitative measurements were assessed by two-sided *t* test and by analysis of variance (ANOVA) with post hoc Tukey test for group differences (for normally distributed measurements) or the Mann-Whitney *U* test and Kruskal-Wallis test with post hoc Dunn-Bonferroni test (for not normally distributed measurements). The Spearman rank correlation was used to assess associations in between the quantitative parameters as well as to early clinical outcome. Positive and negative predictive values (PPV/NPV) and their confidence intervals or, if applicable, logistic regressions were carried out for early clinical outcome and neurological outcome at 3 months. The significance level was set to *p* = 0.05. Means are given with their standard deviation and medians with their interquartile range (IQR), and all confidence intervals are quoted as 95% CI.

## Results

### Patient Characteristics

All included patients (*n* = 38) initially presented with an M1 occlusion and underwent successful mechanical recanalization within a median onset time to treatment (OTT; or time from last seen well to treatment; 12 patients with unknown onset time) of 248 min (interquartile range [IQR] 125–343 min). MRI was acquired in a median of 21 h 36 min (18 h 29 min–24 h 43 min) after mechanical thrombectomy. In patients with significant ipsilateral ICA stenosis (> 70%, *n* = 3), stenosis was treated by stenting during the endovascular procedure.

At the analyzed 24-h time point, four different DWI- and T2/FLAIR-positive infarct patterns within the dependent M1 territory were found: basal ganglia (BG) infarction in 15 patients (39%), infarction mainly in a cortical compartment in 8 patients (21%), BG and cortical infarcts in 7 patients (18%), and mainly small, scattered infarcts in 7 patients (18%). One patient (3%) had no DWI lesion. Seventeen patients (45%) showed HT 24 h after treatment ranging from HI1 to PH1. Further patient characteristics are specified in Table 1.

**Table 1:** Patient characteristics are displayed. If applicable, median and interquartile range are listed

Gender (female/male)	20/18
IV lysis (yes/no)	18/20
Side of occlusion (right/left)	23/15
Age (years)	74 (69–81)
NIHSS at admission	14 (10–17)
Initial ASPECTS	9 (8–10)
Onset time to treatment (min)	248 (125–343)
Recanalization results (TICI 2b/TICI 2c/TICI 3)	9/13/16
Time from recanalization to MRI (hh:mm)	21:36 (18:29–24:43)

### Post-ischemic Perfusion Signatures

**Fig. 1:**
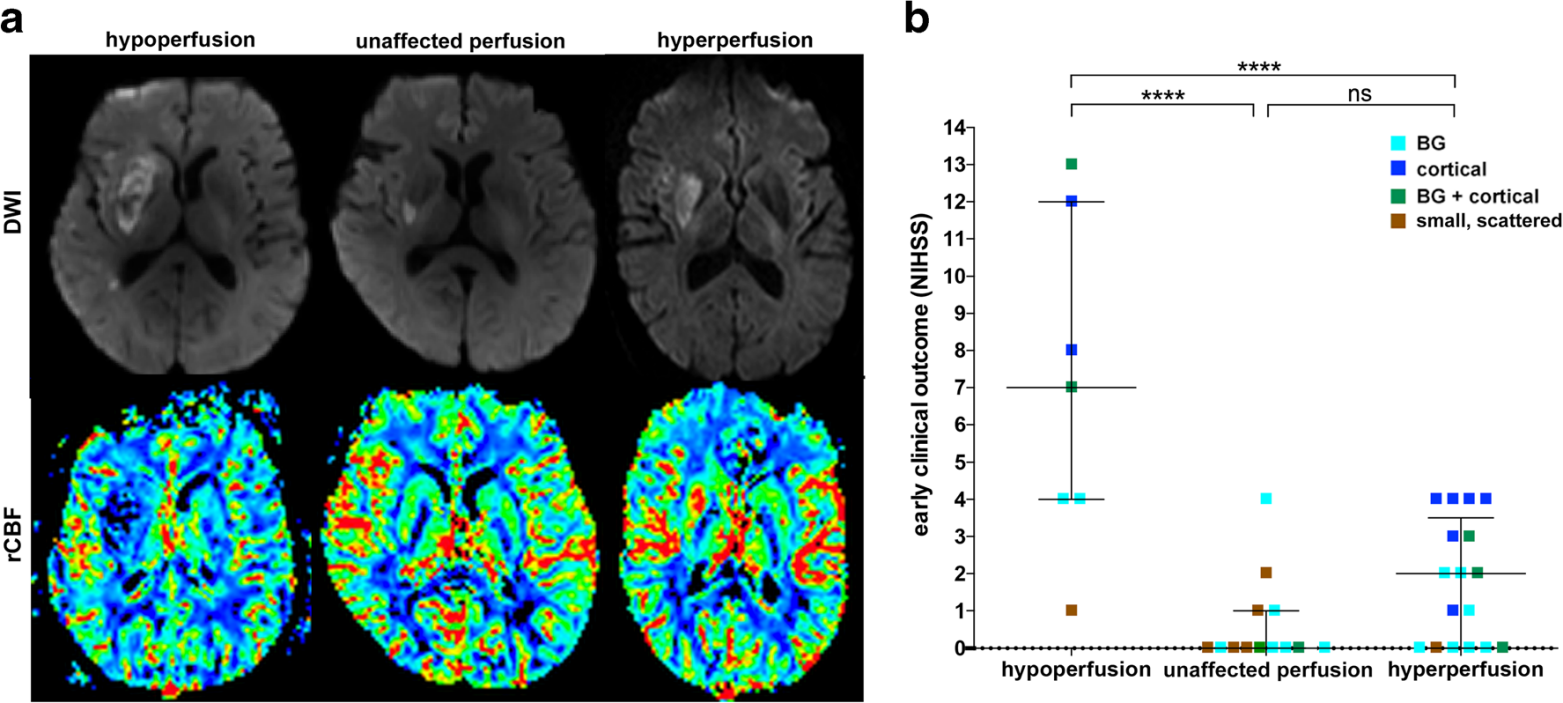
Perfusion signatures illustrated in basal ganglia infarcts. Three representative diffusion-weighted images and corresponding CBF maps are displayed. (a) All three patients show BG infarcts, but different perfusion signatures with hypoperfusion (1st column), unaffected perfusion (2nd column), and hyperperfusion (3rd column). Early clinical outcome (as indicated by NIHSS at discharge from our institute) was worse in patients with hypoperfusion compared to patients with unaffected perfusion or hyperperfusion (b)

Three distinct visual infarct perfusion patterns within the DWI lesion were apparent after successful thrombectomy when compared to the contralateral hemisphere: predominant hypoperfusion (7 of 38 patients, 18%), predominant hyperperfusion (17 of 38 patients, 45%), or unaffected perfusion (13 of 38 patients, 34%) (Fig. 1(a)). One of 38 patients without a DWI lesion 24 h after mechanical recanalization also showed unaffected perfusion. Analysis of variance was significant when comparing mean relative regional CBF (rrCBF) with the visual classification (*p* < 0.001). Median rrCBF was 0.84 (0.76–1.03) in patients with hypoperfusion, while it was 1.36 (1.18–1.41) in the patients with hyperperfusion and 1.06 (0.88–1.11) in the patients with unaffected perfusion. Elevated rrCBF tended to be associated with larger DWI lesion size (*p* = 0.04, *ρ* = 0.34 [0.00–0.68]). HT was not directly related to rrCBF (*p* = 0.69) but instead was observed in both hyperperfusion (52%, 9/17) and hypoperfusion (85%, 6/7), while it was less likely when perfusion was not altered (14%, 2/14). Hyperperfusion was the most frequent perfusion pattern in cortical infarcts (75%, 6/8), as well as in infarcts within BG and cortical location (57%, 4/7) and in infarcts isolated within the BG (46%, 7/15). Unaffected perfusion was most frequently observed in small, scattered infarcts (85%, 6/7) and was further observed in 40% of BG infarcts (6/15) and in 14% of combined BG and cortical infarcts (1/7). Hypoperfusion was present in 25% of cortical infarcts (2/8), in 29% of combined BG and cortical infarct (2/7), and in 13% of BG infarcts (2/15), as well as in 16% of small, scattered infarcts (1/6). Early clinical outcome was significantly better in patients with unaffected perfusion or hyperperfusion compared to patients with hypoperfusion (*p* value < 0.001 and *p* = 0.015 respectively). There was no significant difference in early clinical outcome between patients with either hyperperfusion or unaffected perfusion (*p* value = 0.08) (Fig. 1(b)). Further on, there were no significant interactions between perfusion pattern and diabetes mellitus, arterial hypertension, ipsilateral ICA stenosis (all treated during the endovascular procedure with stenting), or recanalization results (see supplemental table 1).

### Post-ischemic Permeability Signatures

**Fig. 2:**
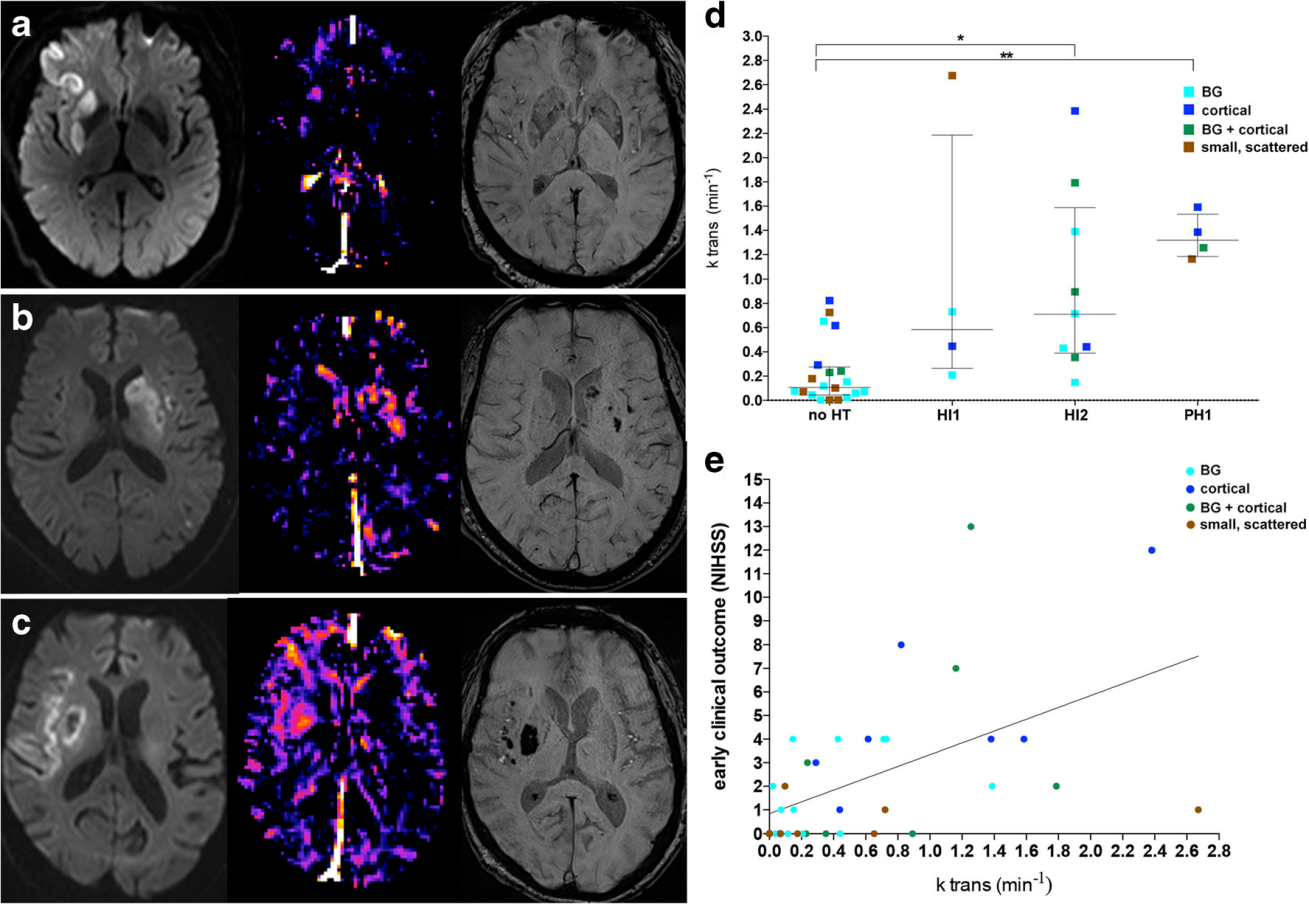
Permeability signatures and their correlation to hemorrhagic transformation illustrated in combined basal ganglia and cortical infarcts. Diffusion-weighted images, *k*_trans_ maps, and susceptibility-weighted images of three representative patients are displayed. In the first row, diffusion restriction in the right striatum and adjacent cortex is seen with focal *k*_trans_ increase in the right striatum, but no hemorrhagic transformation (a). In the second row, diffusion restriction in the left BG and a small portion of the insular cortex as well as in the temporal cortex is evident. Diffusion restriction co-localizes with increased *k*_trans_ and hemorrhagic infarction type 2 in the caudate head in the striatum and the insular cortex. (b) In the third row, diffusion restriction is seen in the right striatum and the adjacent cortex with increased *k*_trans_ and parenchymal hemorrhage type 1 in the striatum as well as petechial hemorrhages in the insular cortex (c). Patients without hemorrhagic transformation displayed a significantly lower *k*_trans_ compared to patients with hemorrhagic infarction type 2 or parenchymal hemorrhage type 1 (d). A moderate correlation of early clinical outcome and *k*_trans_ values was apparent (e)

Blood-brain barrier disruption (BBBD) within the DWI lesions (indicated by elevated *k*_trans_ values) was found in 20 of 38 patients (53%; Fig. 2(a–c)) and was not related to the NIHSS at admission (*p* = 0.66), OTT (*p* = 0.11), mTICI (*p* = 0.51), nor to symptom onset time to MRI (*p* = 0.98). Visual classification of BBBD and quantitative assessment of *k*_trans_ values within the DWI lesion showed excellent agreement (*p* < 0.001). Visually determined BBBD corresponded to a median *k*_trans_ of 0.77 (0.41–1.4)∙10^−3^ min^−1^ within the DWI lesion vs. 0.10 (0.04–0.23)∙10^−3^ min^−1^ in patients without BBBD. *k*_tran*s*_ elevation correlated positively with DWI lesion volume (*p* < 0.001, *ρ* = 0.64 [0.31–0.98]). Furthermore, *k*_trans_ values were significantly increased in patients with HT compared to patients without HT (*p* < 0.001; Fig. 2(d)). BBBD occurred in 86% (6/7) of infarcts in both BG and cortical infarcts, in 63% (5/8) of cortical infarcts, in 47% (7/15) of BG infarcts, and in 29% (2/7) of small, scattered infarcts. We observed worse early clinical outcome in patients with BBBD compared to patients without BBBD (*p* < 0.001, *ρ* = 0.59 (0.25–0.92); Fig. 2(e)). No significant interactions were found between BBBD and risk factors, such as diabetes mellitus, arterial hypertension, or ipsilateral ICA-stenosis, nor between BBBD and recanalization results (see supplemental table 2).

### Combined Post-ischemic Perfusion and Permeability Signatures

**Fig. 3:**
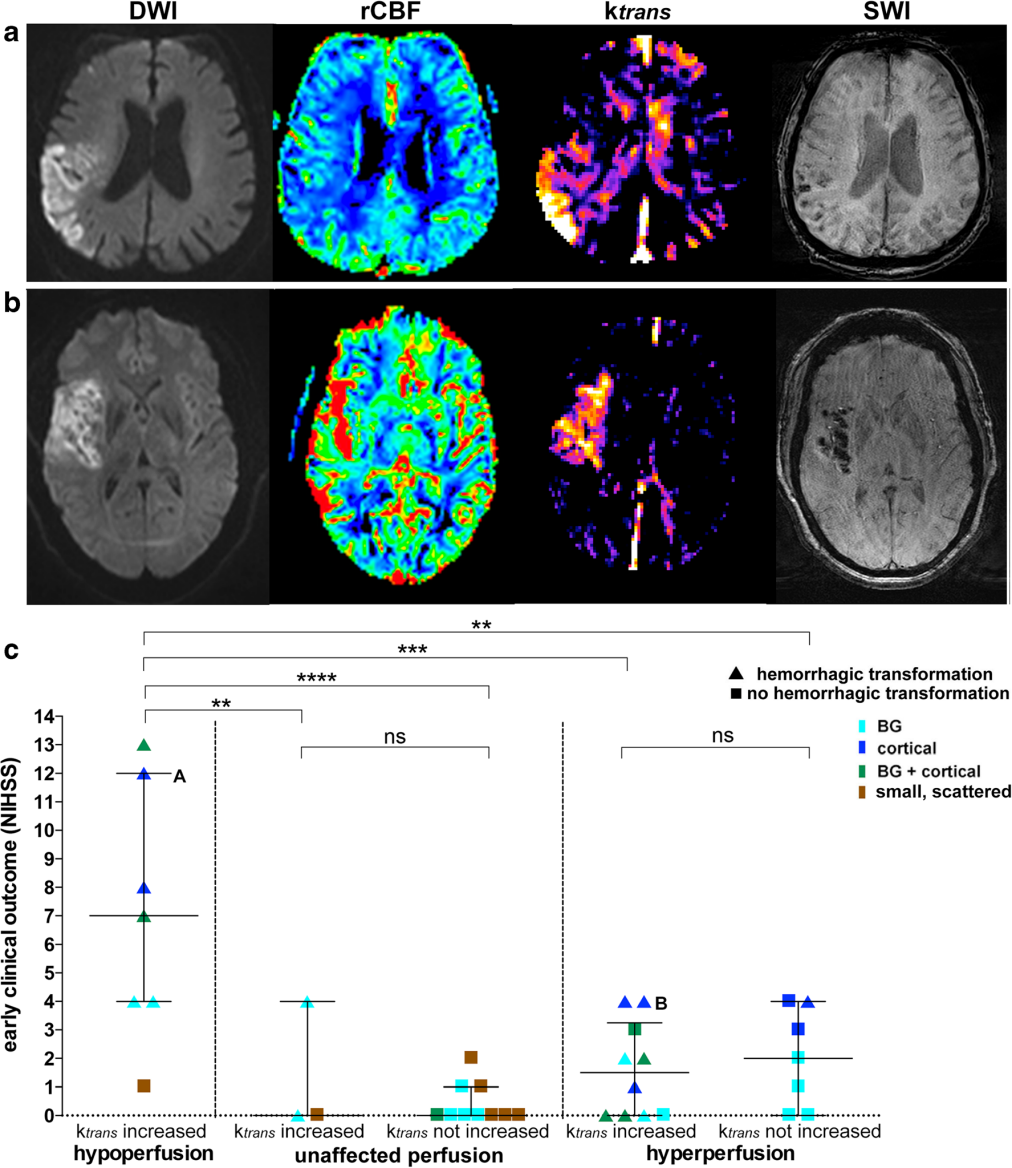
Combined perfusion and permeability signatures illustrated in cortical infarcts. Representative images of two patients with similar diffusion restriction, *k*_trans_ increase, and hemorrhagic transformation, but different perfusion patterns are displayed. The patient in row (a) shows hypoperfusion on the rCBF map, while the patient in row (b) exhibits hyperperfusion. Early clinical outcome shows a marked difference of patient A (NIHSS12) and patient B (NIHSS4). (c) Hemorrhagic transformation is predominantly associated with a *k*_trans_ increase (symbolized by a triangle) and only occurred in one patient without *k*_trans_ increase and hyperperfusion. Early clinical outcome is significantly worse in patients with hypoperfusion compared to all other groups (c)

Visual BBBD was found in all 7 cases with hypoperfusion (100%) and in 10 of 16 cases with hyperperfusion (63%). Respectively, *k*_trans_ elevation in DWI lesions correlated (*p* < 0.001) with both hypoperfusion (*k*_trans_median = 1.16∙10^−3^ min^−1^ [0.77–1.8]) and hyperperfusion (*k*_trans_ median = 0.44∙10^−3^ min^−1^ [0.24–0.89]). In contrast, *k*_trans_ elevation in DWI lesions with unaffected perfusion was less likely (*k*_trans_median = 0.10∙10^−3^ min^−1^ (0.06–0.21), BBBD found in 3/13 patients (23%)). The patient without DWI lesion had no elevated *k*_trans_ either. As HT was strongly associated with BBBD (*p* < 0.001), it was mainly observed in both (*k*_trans_ elevated) hypo- and hyperperfused infarcts (Fig. 3(a, b)). Early clinical outcome was poorer in patients with hypoperfusion (of whom all had BBBD) while it was independent from BBBD in patients with hyperperfusion or unaffected perfusion (Fig. 3(c)).

Also in univariate logistic regression analysis for early clinical outcome, only DWI lesion volume (OR = 0.28 (0.086–0.62), *p* = 0.009) and hypoperfusion (OR = 0.001 (0.00–0.17, *p* = 0.004) were predictors of worse early clinical outcome, while BBBD, hyperperfusion, or unaffected perfusion were not.

### Clinical Outcome After 3 Months

**Fig. 4:**
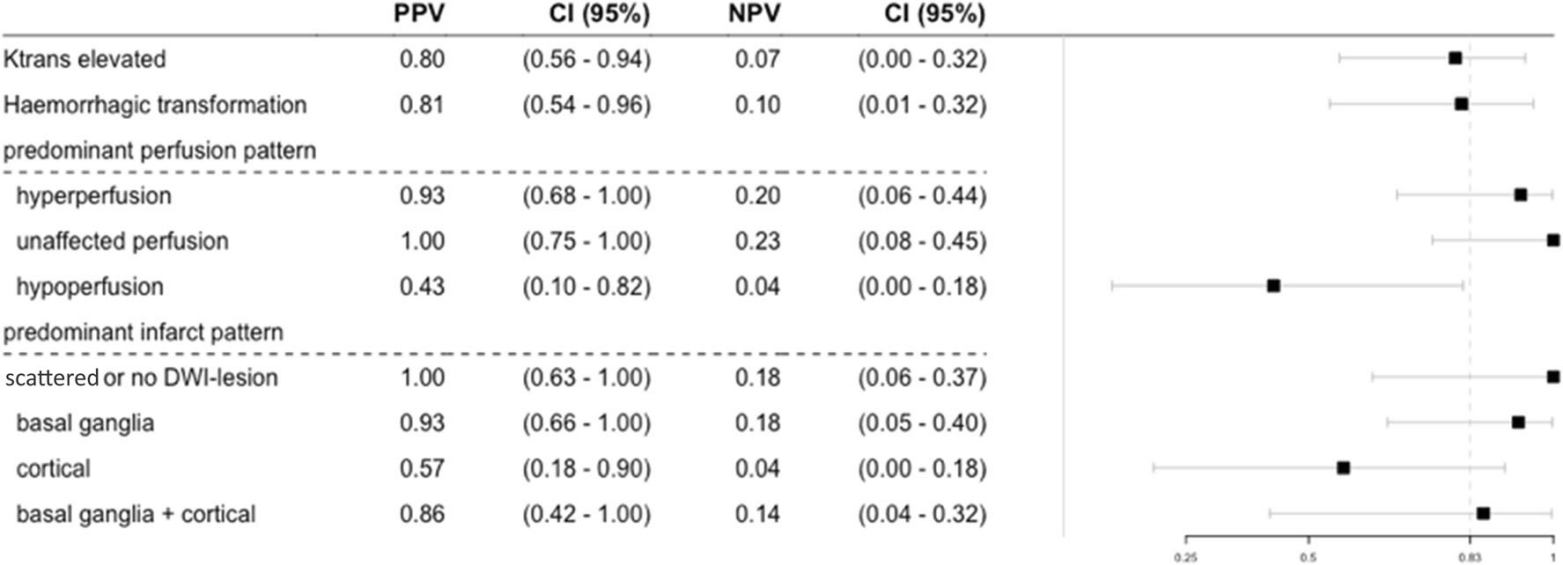
Clinical outcome at 3 months. Positive predictive values (PPV), negative predictive values (NPV), and their confidence intervals for a favorable clinical outcome 3 months after stroke (mRS90 < 3 or mRS90 = pre-stroke mRS = 3). The vertical line within the forest plot indicates the pre-test probability for a favorable clinical outcome in our cohort (0.83). BBBD (as indicated by elevated *k*_trans_) was observed in patients with favorable and in patients with unfavorable clinical outcome. Moreover, it was also observed in both hyperperfusion and (in all cases of) hypoperfusion, with only the latter being associated with poorer clinical outcome at 3 months

Favorable clinical outcome 3 months after stroke was achieved in 31 of 36 patients (83%). The mRS90 values of two patients were not included as one patient’s mRS90 was not available and one patient died from a previous disease despite excellent early clinical recovery after mechanical thrombectomy. These patients were excluded from the final outcome analysis to correlate clinical outcome directly with the brain tissue response. Hence, the pre-test probability for a favorable clinical outcome was 0.83 in our cohort (Fig. 4). With a positive predictive value (PPV) of 0.8 (0.56–0.94) and an OR of 0.43 (0.12–1.46, *p* = 0.17), BBBD or *k*_trans_ elevation did not predict for favorable clinical outcomes. As no symptomatic hemorrhage occurred in our cohort, the same applied to HT (PPV = 0.81 [0.56–0.94], OR = 0.48 [0.06–3.3], *p* = 0.46). However, while BBBD was observed in patients with hyperperfusion, unaffected perfusion, and (all cases of) hypoperfusion, only the latter was associated with poorer clinical outcome at 3 months (PPV = 0.43 [0.10–0.82]) in comparison to hyperperfusion or unaffected perfusion (OR = 0.03 [0.001–0.26], *p* = 0.005). A favorable clinical outcome was more likely in patients with hyperperfusion (PPV = 0.93 [0.68–1.0]) or unaffected perfusion (PPV = 1.0 [0.75–1.0]).

All patients with only small, scattered infarcts (or in 1 patient with no DWI lesion) had a favorable clinical outcome at 3 months (PPV = 1.0 [0.63–1]), while a cortical infarct pattern had a PPV of only 0.57 [0.18–0.90]. Lastly, as acute ASPECTS scores on pre-interventional imaging were relatively homogenous in our cohort (median baseline ASPECTS of 9 [8–10]), baseline ASPECT score was not significantly associated with clinical outcome (OR 1.63 [0.74–4.05], *p* = 0.24), while DWI lesion size on follow-up imaging was associated with clinical outcome (OR per 10 ml increase: 0.43 [0.18–0.88], *p* = 0.027).

## Discussion

By studying MRI perfusion and permeability infarct patterns within 24 h after mechanical thrombectomy, we identified distinct post-ischemic perfusion and permeability signatures within the DWI lesion. Since only patients with successful recanalization were included in the study, our study design allows to particularly focus on the post-ischemic tissue status itself with regard to microvascular perfusion and radiologic BBBD in the absence of persistent macrovascular occlusion.

Prior studies have shown a strong correlation between infarct size on follow-up imaging and clinical outcome [23]. In addition to infarct size, we present hypoperfusion after successful recanalization as an independent predictor for an unfavorable clinical outcome. This is in line with previous studies, and, as we only included patients after successful mechanical thrombectomy, this is independent of restored macrovasculature [24, 25]. Hypoperfusion despite successful large-vessel recanalization is thought to result from insufficient capillary reflow in irreversibly injured tissue, induced e.g. by post-ischemic aggregation of blood elements including microthrombi [26, 27]. The administration of i.v. tPA has been shown to improve microvascular perfusion, especially in areas distant to the core infarct [28]. As we analyzed perfusion and permeability patterns within the DWI lesion, we did not observe significant interactions between perfusion and permeability patterns and administration of i.v. tPA in our study.

Macrovascular revascularization (i.e., mechanical thrombectomy) with hypoperfusion at the microvascular level is associated with HT [29]. This finding is emphasized by our finding that all patients with hypoperfused DWI lesions exhibited radiologic BBBD and all except one patient developed HT. We point out that the association of BBBD with HT 1 day after stroke does not allow us to conclude whether HT is caused by BBBD or vice versa as we even observed BBBD without HT.

Radiologic BBBD was seen in all cases of hypoperfusion and also in 60% of patients with hyperperfusion. In our study, hyperperfusion was associated with favorable clinical outcome independent from the occurrence of BBBD, implying that hyperperfused tissue early after successful recanalization may recover or at least show less infarct progression compared to hypoperfused tissue. Hyperperfusion has been associated with less severely injured tissue before [25, 30] but has been linked to both favorable and unfavorable clinical outcomes after stroke [10, 31]. While early hyperperfusion has been associated with tissue survival [32], tissue with late hyperperfusion or more severe prior ischemic injury has a higher likelihood to turn into infarction [31, 33].

Hyperperfusion occurs frequently after successful recanalization and may preserve tissue that is not irreversibly damaged [11, 14]. We observed hyperperfusion in 45% of our patients, which is 5–20% higher than reported in cohorts with both complete and incomplete recanalization [11–13, 34], but comparable to subgroups with successful recanalization within these studies (47–48%). Animal studies have demonstrated early on that recirculation after arterial occlusion induces abrupt tissue hyperperfusion, which has been considered as proof of successful reperfusion in tissue of prior ischemia [35, 36]. The underlying mechanisms of hyperperfusion are not clear but felt to come from the loss of cerebrovascular autoregulation with consecutive vasodilation and the release of vasodilating substances. Additionally, neurogenically mediated vessel dilation has been shown. Hyperperfusion is considered a non-nutritional “luxury perfusion,” but with time, there is the potential of the hyperperfusion to normalize [31, 37, 38].

Previous studies showed hyperperfusion to correlate with a higher risk for HT and poorer clinical outcome after stroke [12, 13]. By combining perfusion and permeability imaging in our study, we show that HT is not primarily related to hyperperfusion, but to BBBD. However, even though BBBD and hyperperfusion represent different pathophysiological mechanisms, they may still be linked. As BBBD is caused by ischemia-related damage to the neurovascular unit, it disturbs capillary flow and can cause shunting of large volumes of blood through the surrounding capillary bed, which in turn can result in restored or increased net tissue perfusion [39].

Most studies of BBBD in stroke have assessed patients before treatment and found an association of BBBD with both an increased risk of hemorrhage and poorer clinical outcome [40–42]. Moreover, increasing evidence suggests that the severity of early BBBD not only reflects the ischemic damage in the acute phase but also determines the later second phase of inflammatory BBBD [43–45].

We underline that in our study cohort, BBBD was not an independent predictor of neurological outcome. Instead, *k*_trans_ elevation was directly associated with DWI lesion size in our study, with only the latter being a predictor of clinical outcome. While this could be due to the small group size and the occurrence of no symptomatic hemorrhages within our study population, similar results were obtained by a recent DCE-MRI study that found no correlation between elevated *k*_trans_ values after stroke treatment and clinical outcome after 3 months [46]. Moreover, we observed HT in one patient without BBBD 24 h after stroke, raising the possibility that BBBD may already have reversed. The results motivate for further studies on the important question of temporal evolution of BBBD and respectively microvascular impairment after restored perfusion.

We recognize the limitations of our study which could be addressed in further studies, including the relatively small group size (*n* = 38), the single-center study design, the fact that patients had to give written consent before study inclusion (and thus selecting towards smaller infarct volumes and thus better clinical outcomes), and that our study imaged only at one time point after stroke. More elaborate neurological tests at 3 months may additionally improve outcome correlation and interpretation of the observed perfusion and permeability patterns in future studies.

## Conclusions

So far, the differentiation between upstream macrovascular flow and the downstream brain tissue response of microvascular perfusion and permeability has been particularly challenging. By studying T2* DSC perfusion and DCE T1 permeability stroke signatures using the most appropriate and established method within the clinical setting, we reveal distinct post-ischemic pathophysiological tissue responses in individual patients. Hypoperfusion after macrovascular recanalization was an independent predictor for unfavorable clinical outcome and occurred only in combination with BBBD, indicating irreversible ischemic injury. In contrast, patients with hyperperfusion had good clinical outcomes with less severe tissue damage, irrespective of BBBD. BBBD alone did not discriminate between favorable and unfavorable outcomes in patients after successful thrombectomy. Combined perfusion and permeability imaging therefore may help to differentiate between upstream macrovascular flow restoration and downstream microvascular perfusion and ischemia-related brain tissue damage. These imaging methods may help to identify patients who benefit from adjunctive, neuroprotective stroke therapies to reduce microvascular damage and consecutive brain injury progression.

## Supplementary Information

ESM 1 (DOCX 23 kb)

## Data Availability

Data will be made available on reasonable request.
