# Supplementary material for: Combined Perfusion and Permeability Imaging Reveals Different Pathophysiologic Tissue Responses After Successful Thrombectomy
Source: Transl Stroke Res. 2021 Jan 11;12(5):799–807. doi: 10.1007/s12975-020-00885-y (PMC8421283; doi:10.1007/s12975-020-00885-y)
Supplement: Supplementary file 1 — (DOCX 23 kb) [file 12975_2020_885_MOESM1_ESM.docx]

**Supplemental table 1:** Perfusion patterns in relation to risk factors, recanalization score and HT

|  | Hypoperfusion  (n of n = 7, %) | unaffected perfusion  (n of n = 14*, %) | Hyperperfusion (n of n = 17, %) |
| --- | --- | --- | --- |
| Diabetes mellitus | 2 (29 %) | 3 (21 %) | 1 (6 %) |
| Hypertension | 7 (100 %) | 9 (64 %) | 15 (88 %) |
| ACI-Stenosis (treated) | 2 (29 %) | 0 (0 %) | 1 (6 %) |
|  | | | |
| iv tPA | 5 (71 %) | 6 (43 %) | 9 (56 %) |
|  | | | |
| TICI 2b | 2 (29 %) | 4 (29 %) | 3 (18 %) |
| TICI 2c | 3 (43 %) | 5 (36 %) | 5 (29 %) |
| TICI 3 | 2 (29 %) | 5 (36 %) | 9 (53 %) |
|  | | | |
| no HT | 1 (1 iv tPA) | 12 (4 iv tPA) | 8 (3 iv tPA) |
| HI 1 | 1 (no iv tPA) | 1 (1 iv tPA) | 2 (2 iv tPA) |
| HI 2 | 3 (3 iv tPA) | 1 (1 iv tPA) | 5 (4 iv tPA) |
| PH 1 | 2 (1 iv tPA) | 0 | 2 (1 iv tPA) |
| PH 2 | 0 | 0 | 0 |

*One of 14 patient did not show a DWI lesion 24h after mechanical recanalization.

**Supplemental table 2:** Permeability patterns in relation to risk factors, recanalization score and HT

|  | *k*_trans_ increased  (n of n = 20, %)) | *k*_trans_ not increased  (n of n = 18*, %) |
| --- | --- | --- |
| Diabetes mellitus | 4 (20 %) | 2 (5 %) |
| Hypertension | 18 (90 %) | 13 (72 %) |
| ACI-Stenosis (treated) | 2 (5 %) | 1 (4 %) |
|  | | |
| iv tPA | 13 (65 %) | 7 (39 %) |
|  | | |
| TICI 2b | 5 (25 %) | 4 (22 %) |
| TICI 2c | 8 (40 %) | 5 (28 %) |
| TICI 3 | 7 (35 %) | 9 (50 %) |
|  | | |
| no HT | 4 (2 iv tPA) | 17 (7 iv tPA) |
| HI 1 | 3 (2 iv tPA) | 1 (no iv tPA) |
| HI 2 | 9 (8 iv tPA) | 0 |
| PH 1 | 4 (1 iv tPA) | 0 |
| PH 2 | 0 | 0 |

*One of 18 patient did not show a DWI lesion 24h after mechanical recanalization.
